# A case report of bacteremia manifesting as an overwhelming postsplenectomy infection due to *Streptococcus pneumoniae* post vaccination

**DOI:** 10.1186/s40792-016-0173-2

**Published:** 2016-05-25

**Authors:** Kosuke Hirose, Hirohisa Okabe, Tomoharu Yoshizumi, Hideaki Uchiyama, Toru Ikegami, Norifumi Harimoto, Shinji Itoh, Koichi Kimura, Hideo Baba, Yoshihiko Maehara

**Affiliations:** Department of Surgery and Science, Graduate School of Medical Sciences, Kyushu University, Maidashi 3-1-1, Higashi-Ku, Fukuoka, 812-8582 Japan; Department of Gastroenterological Surgery, Graduate School of Life Sciences, Kumamoto University, Fukuoka, Japan

**Keywords:** Overwhelming postsplenectomy infection, *Streptococcus pneumoniae*, Spontaneous bacterial peritonitis

## Abstract

A 62-year-old woman was admitted for acute epigastralgia and high-grade fever of over 39 °C. The patient had undergone splenectomy for idiopathic portal hypertension 1 year ago and vaccination against *Streptococcus pneumoniae* immediately post operation. She developed localized peritoneal irritation and abdominal distension. Her serum creatinine had increased to 1.5 mg/dL and procalcitonin was 12.5 ng/ml. Computed tomography of the abdomen revealed edematous large intestine and increased ascites. From these results, the patient was considered to have spontaneous bacterial peritonitis (SBP). Vancomycin (VCM) and doripenem (DRPM) were administered to control the infection. Unexpectedly, *S. pneumoniae* was detected in the blood culture. Hence, ampicillin/sulbactam was administered after discontinuing VCM. The patient recovered without any life-threatening complications and was discharged after 10 days. In conclusion, overwhelming postsplenectomy infection (OPSI) due to *S. pneumoniae* could develop in patient with splenectomy even after vaccination. Although the bacteremia probably due to SBP and acute renal dysfunction was accompanied by OPSI, our patient recovered rapidly.

## Background

Overwhelming postsplenectomy infection (OPSI) is a serious disease with a high mortality rate, especially in patients with hematopoietic diseases [1]. Splenectomized patients are at a risk of developing life-threatening sepsis. Severe sepsis develops due to several risk factors such as young age at the time of splenectomy [2], short time interval from splenectomy [3], hematological disorders that necessitate splenectomy [4], and the overall immune status of the patient. Some patients with hematologic disorders fail to acquire immune protection by vaccination against *Staphylococcus pneumoniae* because the underlying severe hematological disease associated with immunosuppression inhibits proper production of immunoglobulins. However, to our knowledge, there have been no reports of patients without any impairment in the immune response who have developed OPSI after vaccination.

Herein, we present a rare case of bacteremia probably due to spontaneous bacterial peritonitis (SBP) caused by *S. pneumoniae* after splenectomy and subsequent vaccination.

## Case presentation

**Fig. 1:**
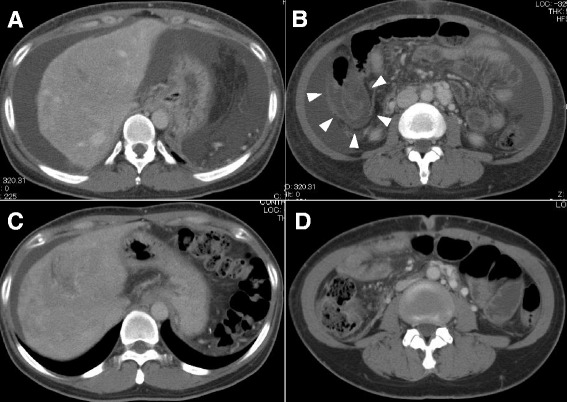
Computed tomography (CT) at the onset of spontaneous bacterial peritonitis and after discharge. **a**, **b** CT images obtained at admission. Ascites (**a**) and remarkably thickened large intestinal wall (*arrowhead*) were confirmed. **c**, **d** CT images obtained 1 1/2 month after discharge. Ascites had decreased (**c**) and the wall thickness of the large intestine appeared normal (**d**)

A 62-year-old woman was admitted for acute epigastralgia and high-grade fever of over 39 °C. The patient developed abdominal distension and pitting edema on bilateral lower extremities, and showed signs of localized peritoneal irritation on admission. The laboratory data was as follows: white blood cell count, 22.29 × 10^3^/μL; serum creatinine, 1.55 mg/dL; prothrombin time (PT) activity, 21 %; and C-reactive protein, 7.57 mg/dL (Table 1). She was diagnosed with idiopathic portal hypertension 10 years ago and had undergone splenectomy a year ago. Portal thrombosis was observed on computed tomography (CT) a week after splenectomy, and anticoagulant (warfarin) therapy was administered. The patient had received vaccination against *S. pneumoniae* after the operation. PT activity was controlled around 50 % before the admission. Enhanced CT on admission showed increased ascites (Fig. 1a) and edematous changes in the large intestine (Fig. 1b). The portal vein was patent, and thrombosis in the portal vein persisted. On the basis of the symptoms and the radiological findings, the patient was considered to have SBP, and doripenem (1.5 g/day) and vancomycin (VCM, 1.5 g/day) were administered. Although we did not perform the bacterial examination on ascites on admission, *S. pneumoniae* was detected in the blood culture on the following day. Hence, VCM was discontinued and ampicillin/sulbactam (6 g/day) was administered. Antibiotic treatment was maintained for 5 days, and the symptoms disappeared a week after the treatment. Following this, the patient was started on levofloxacin (500 mg/day) and discharged 10 days after the admission. Creatinine levels improved to the pre-admission levels (0.85 mg/dL) and the PT activity increased to 60 %, leading to re-administration of warfarin. CT obtained a month after the discharge showed that ascites had decreased and the abnormal findings of the large intestine had disappeared (Fig. 1c, d).

**Table 1:** Laboratory data on admission

WBC	22.29	×10^3^/μL	Albumin	1.9	mg/dL
RBC	3.37	×10^6^/μL	BUN	27	mg/dL
Hemoglobin	10.9	g/dL	Creatinine	1.55	mg/dL
Hematocrit	33.2	%	Amylase	50	U/L
Platelet	177	×10^3^/μL	Na	135	mEq/L
PT activity	40.3	%	K	3.3	mEq/L
AST	23	U/L	Cl	98	mEq/L
ALT	15	U/L	CRP	7.57	mg/dL
ALP	223	U/L	CK	90	U/L
LDH	223	U/L	NH_3_	77	μg/dL
γ-GTP	9	U/L	Procalcitonin	12.51	ng/mL
Total bilirubin	1.4	mg/dL	Endotoxin	<1.852	
Total protein	5.2	mg/dL			

*WBC* white blood cell, *RBC* red blood cell, *PT* prothrombin time, *AST* aspartate aminotransferases, *ALT* alanine aminotransferases, *ALP* alkali phosphatase, *LDH* lactate dehydrogenase, *γ-GTP* γ-glutamyltransferase, *BUN* blood urea nitrogen, *CRP* C-reactive protein, *CK* creatinine kinase, *NH*_*3*_ ammonia

### Discussion

OPSI is characterized by severe distinct or concomitant infections causing pneumonia, meningitis, SBP, and sepsis. The clinical course progresses to coma and death within 24–48 h, due to the high incidence of shock, hypoglycemia, marked acidosis, electrolyte abnormalities, respiratory distress, and disseminated intravascular coagulation [5, 6]. Without the spleen, prompt antibody production against a new antigen is impaired and rapid bacteria proliferation causes increased infection rate of pneumococcal diseases (12–25 times greater than in the population at large) [7].

SBP that occurs in patients with liver cirrhosis has some well-known disease-specific mechanisms. Based on the clinical observations, patients with portal hypertension are prone to develop bacterial translocation that conceivably causes SBP. This translocation is supported by increased levels of lipopolysaccharide which is a surrogate parameter of gram-negative bacteremia [8], bacterial overgrowth in the small bowel [9], and decreased barrier function of the intestine in patients with liver cirrhosis [10]. Our patient had portal hypertension that increased the probability of SBP irrespective of splenectomy. Notably, previous investigations on a substantial number of patients with cirrhosis and ascitic fluid infection revealed that SBP in the patients showed high mortality, and *Escherichia coli* (61.3 %) and *S. pneumoniae* (11.3 %) were the most causative organisms. Although we hesitated to draw ascites for bacterial examination due to high risk of intraperitoneal bleeding at the puncture, it turned out that the patient had bacteremia by blood culture test performed on admission. It was found that increased creatinine >1.1 mg/dL and a positive blood culture were independent factors involved in mortality [11]. Our patient had both these factors; however, she recovered without any complications.

To our knowledge, there are no studies conducted on the occurrence of OPSI after vaccination, except one that focused on patients with hematologic disorders [1]. In our case, we considered two probabilities. First, appropriate vaccination might have decreased the severity of OPSI resulting in rapid and remarkable recovery from bacteremia caused by *S. pneumoniae*. The patient had no immune response-related disorder, although the vaccination was performed after splenectomy. Second, failure of the vaccination might have caused severe infectious state by *S. pneumoniae*. However, the latter is unlikely to occur in the current case because the patient was not under immunosuppressive state. Patients with cirrhosis or portal hypertension might need a careful follow-up after splenectomy, and a large-scale cohort study of patients with cirrhosis who have undergone splenectomy is necessary to clarify this issue.

## Conclusions

We encountered a rare case of bacteremia with OPSI caused by *S. pneumoniae* 1 year after splenectomy and subsequent vaccination. However, she recovered without any complications. OPSI due to *S. pneumoniae* even after vaccination should be suspected in patients with splenectomy.
